# Robotic‐Assisted Cytoreductive Surgery and Hyperthermic Intrathoracic Chemotherapy in Metastatic Pleuropulmonary Leiomyosarcoma: A Case Report

**DOI:** 10.1155/crom/4409000

**Published:** 2026-01-02

**Authors:** Mariana Canevari de Oliveira, Paula Duarte D’Ambrosio, David Pinheiro Cunha, Rodrigo Carvalho Marotta, Marcelo Manzano Said, Ricardo Mingarini Terra

**Affiliations:** ^1^ Division of Thoracic Surgery in Hospital da PUC-Campinas, Campinas, State of São Paulo, Brazil; ^2^ University of São Paulo, São Paulo, State of Sao Paulo, Brazil, usp.br; ^3^ Grupo SOnHe, Campinas, State of Sao Paulo, Brazil; ^4^ Instituto do Radium de Campinas, Campinas, State of Sao Paulo, Brazil

**Keywords:** HITHOC, hyperthermic intrathoracic chemotherapy, leiomyosarcoma, pleural metastases, robotic surgery, thoracic oncology

## Abstract

Leiomyosarcoma (LMS) is a rare and aggressive soft tissue sarcoma known for its propensity for hematogenous dissemination, frequently involving the lungs and pleura. We present a case of metastatic pleuropulmonary LMS managed with a novel combined approach: robotic‐assisted cytoreductive surgery and hyperthermic intrathoracic chemotherapy (HITHOC). A 61‐year‐old male with high‐grade LMS developed progressive pleural and pulmonary metastases following prior systemic chemotherapy and palliative radiotherapy. Diagnostic imaging showed enlarging pleural nodules and diaphragmatic thickening, with PET‐CT confirming intense hypermetabolic activity confined to the right hemithorax. A multistep robotic‐assisted thoracic procedure was performed, including total pleurectomy, pulmonary segmentectomy, mediastinal lymphadenectomy, and HITHOC utilizing cisplatin at 42°C. The patient achieved an uncomplicated postoperative course and reported complete symptomatic relief of pre‐existing pleuritic pain. At 6‐months postprocedure, follow‐up imaging confirmed isolated local recurrence (two subpleural nodules). However, no regional lymphadenopathy or distant progression was detected. The patient was transitioned to pazopanib therapy, which resulted in radiological stability and maintained symptomatic control through the 12‐month postoperative follow‐up period. This case demonstrates the feasibility, safety, and therapeutic value of robotic cytoreduction with HITHOC in selected metastatic LMS, achieving complete macroscopic resection and sustained 12‐month disease control when integrated with targeted therapy.

## 1. Introduction

Leiomyosarcoma (LMS) is a malignant tumor of mesenchymal origin with high metastatic potential, frequently disseminating to the lungs and pleura. Although systemic chemotherapy remains the mainstay for advanced LMS, durable disease control is rarely achieved [1]. In selected patients with oligometastatic or localized progressive disease, aggressive local treatment can provide meaningful symptom relief and improved locoregional control [2].

Robotic‐assisted thoracic surgery (RATS) has markedly advanced the execution of complex thoracic oncology procedures. Its enhanced dexterity and three‐dimensional visualization enable precise cytoreduction in anatomically challenging pleural regions. These technical advantages translate into reduced postoperative pain and faster recovery—critical benefits for patients undergoing multimodal treatments [3].

Hyperthermic intrathoracic chemotherapy (HITHOC) allows direct perfusion of heated chemotherapy within the pleural cavity, achieving high local drug concentrations with synergistic hyperthermic cytotoxicity while minimizing systemic toxicity [4]. Recent studies support the integration of RATS with HITHOC, suggesting its feasibility and favorable safety profile in primary and secondary pleural malignancies [5].

Although definitive evidence supporting this specific combined modality remains limited in the setting of sarcoma, this integrated approach represents a feasible therapeutic strategy that aligns with current trends toward minimally invasive, function‐preserving oncologic surgery [6].

We report a challenging case of pleuropulmonary metastatic LMS managed with robotic‐assisted cytoreduction and HITHOC, illustrating the potential of this multimodal framework to achieve essential symptomatic relief and temporary disease stabilization within a comprehensive treatment plan.

## 2. Case Presentation

A 61‐year‐old male was initially diagnosed with high‐grade LMS originating in the right upper limb, for which he underwent marginal resection. During surveillance, pulmonary and pleural metastases were identified. The patient received systemic doxorubicin‐based chemotherapy and palliative thoracic radiotherapy for pain control. However, subsequent imaging demonstrated disease progression, characterized by enlarging posterior pleural nodules and diffuse diaphragmatic thickening.

**Figure 1:**
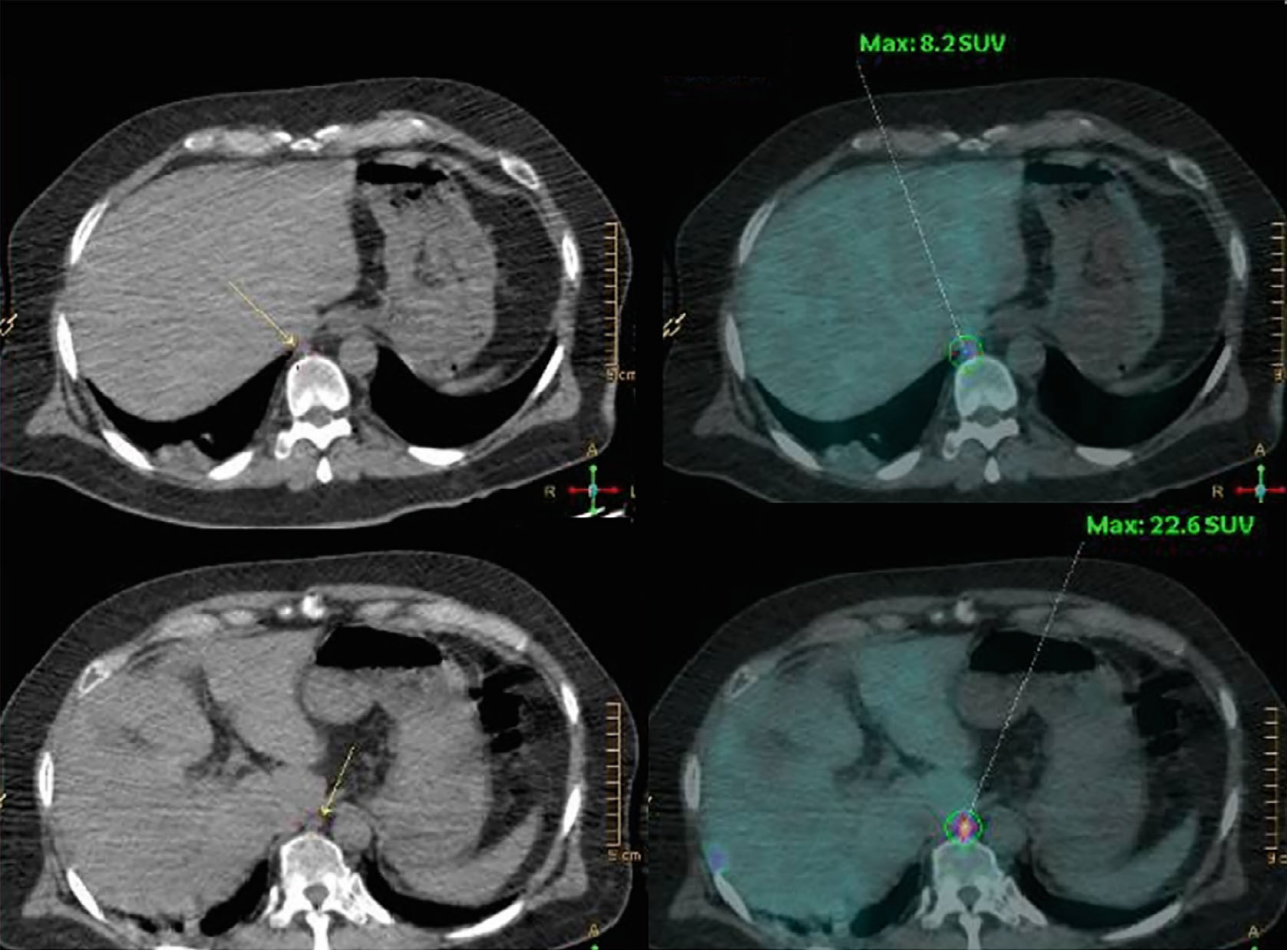
Preoperative PET‐CT demonstrated increased 18F‐FDG uptake in multiple pleuropulmonary and diaphragmatic lesions, including right subpleural nodules (SUVmax up to 33.9), a right upper lobe posterior lesion, a hypermetabolic right phrenic lymph node (SUV 22.6), and nodular thickening of the right diaphragm (SUVs 27.9 and 32.8), consistent with metabolically active metastatic disease.

18F‐FDG PET‐CT revealed intense hypermetabolic uptake within pleural and diaphragmatic nodules (SUVmax up to 33.9) and a right phrenic lymph node (SUV 22.6), which confirmed metabolically active disease confined to the right hemithorax, without evidence of extrathoracic dissemination (Figure 1).

Robotic‐assisted cytoreduction was subsequently performed using the Da Vinci Xi surgical platform. The procedure included a total parietal pleurectomy to remove multiple posterior and diaphragmatic pleural nodules, complemented by a segmentectomy involving three basal segments of the right lower lobe and mediastinal lymphadenectomy. The phrenic nerve was successfully preserved, and no macroscopic residual disease was observed at the completion of resection. Immediately thereafter, HITHOC was administered via a closed perfusion circuit using cisplatin (100 mg/m^2^) at 42°C for 60 min. The postoperative course was uneventful. The patient reported complete resolution of pleuritic pain and was discharged on postoperative Day 6.

At the 3‐month follow‐up, the patient remained asymptomatic. A contrast‐enhanced computed tomography (CT) scan performed at 4 months postoperatively demonstrated only expected postsurgical changes, with no evidence of recurrence (Figure 2, side‐by‐side comparison of pre‐ and postoperative images).

Figure 2Side‐by‐side comparison of pre‐ and postoperative contrast‐enhanced chest CT images. Postoperative images demonstrate changes after pleurectomy and segmentectomy, with no evidence of pleural thickening, nodular recurrence, or residual disease. A small loculated pleural effusion is visible, consistent with expected postoperative findings.

**Figure:**
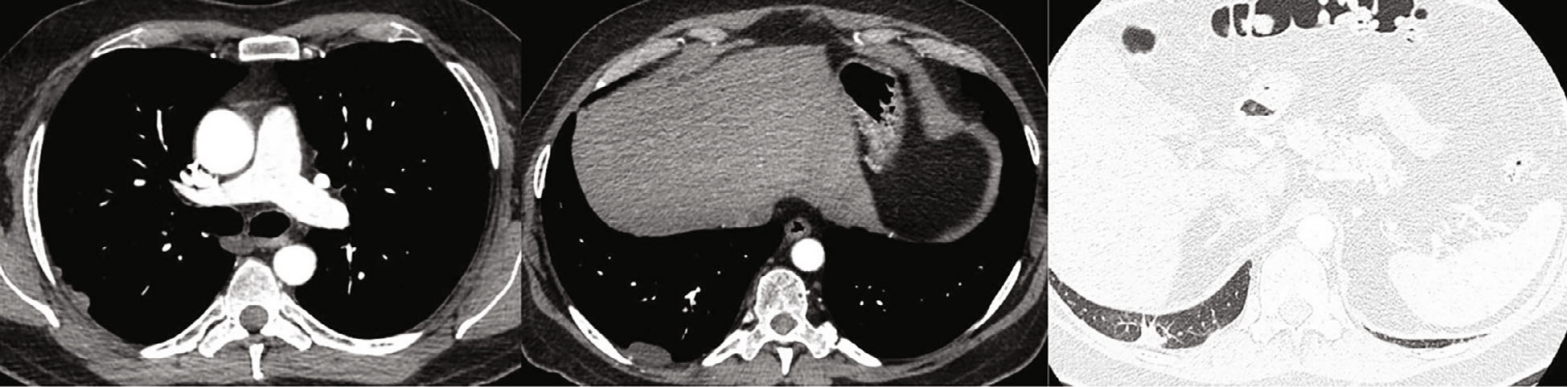
(a) Preoperative chest computed tomography

**Figure:**
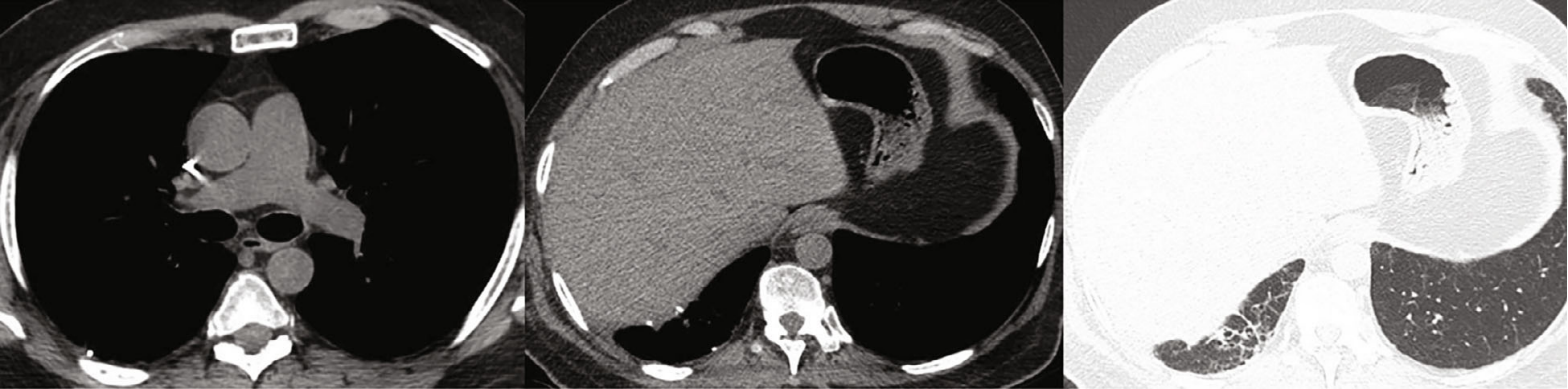
(b) Postoperative chest computed tomography

At 6 months postoperatively, surveillance PET‐CT demonstrated two hypermetabolic subpleural nodules in the right lower lobe (SUVs 4.1 and 6.9), consistent with local recurrence, without regional lymph node or distant metastatic disease. The patient reported sustained improvement in pleuritic pain but declined immediate reoperation. Pazopanib (800 mg daily) was initiated, resulting in radiologic stability over the following months.

At 12 months postoperatively, follow‐up CT imaging demonstrated stable pleural nodular thickening in the right lower lobe and a small ipsilateral pleural effusion, findings consistent with continued disease control under pazopanib therapy.

## 3. Discussion

This case reinforces the feasibility, safety, and potential clinical benefits of combining robotic‐assisted cytoreduction with HITHOC in metastatic LMS, even in patients with prior exposure to systemic chemotherapy and radiotherapy. The robotic platform provided the necessary dexterity and high‐definition visualization to achieve complete resection of pleural and diaphragmatic metastases while preserving critical structures. This resulted in an uncomplicated postoperative course and rapid patient recovery. These features are consistent with the increasing body of evidence that robotic approaches can expand the indications for complex intrathoracic resections while maintaining low morbidity [3, 7].

HITHOC, when applied in conjunction with cytoreductive surgery, enhances local tumor control by targeting microscopic residual disease. Recent reviews and multicenter experiences have shown promising outcomes for HITHOC in both primary and secondary pleural malignancies, including sarcoma, with acceptable toxicity and improved progression‐free survival in selected patients [5, 8].

Although recurrence occurred at 6 months, this interval aligns with the expected disease trajectory in metastatic LMS and underscores the transient but meaningful benefit of cytoreductive surgery for quality‐of‐life enhancement and local symptom control. Importantly, the combination of robotic surgery and HITHOC provided a window of pain‐free, disease‐free survival that facilitated early initiation of targeted therapy.

From a prognostic standpoint, studies of pulmonary metastasectomy in soft‐tissue sarcoma report median relapse‐free survival around 13 months, with disease‐free intervals shorter than 12 months identified as adverse but not uncommon [9]. Therefore, this case′s evolution is consistent with the natural history of advanced LMS rather than an early failure [2]. The subsequent stability achieved with pazopanib mirrors the outcomes observed in the PALETTE trial, where median progression‐free survival reached 4.6 months [10].

Overall, this case supports a proactive surgical approach in selected patients with pleuropulmonary LMS, demonstrating that robotic cytoreduction combined with HITHOC can provide effective short‐term control, meaningful symptom relief, and a foundation for integrated multimodal therapy. The minimally invasive nature of the robotic platform may further enable repeated interventions or salvage procedures with reduced morbidity, extending the therapeutic possibilities for this challenging disease [11, 12].

The present case has limitations. Initial follow‐up was limited to 4 months, and the documented 6‐month local recurrence emphasizes the necessity of prolonged surveillance. Nonetheless, the procedure achieved both symptomatic and temporary oncologic control, confirming its role within a multidisciplinary management strategy.

Future studies should focus on defining patient selection criteria and long‐term outcomes of robotic‐assisted HITHOC, potentially establishing this combined approach as a standard option for locoregional control in metastatic sarcoma.

## Ethics Statement

Written informed consent was obtained from the patient for the publication of this case report and images. Institutional review board approval was not required for this case report, in accordance with local regulations.

## Conflicts of Interest

The authors declare no conflicts of interest.

## Funding

No funding was received for this manuscript.

## Data Availability

The data that support the findings of this study are available on request from the corresponding author. The data are not publicly available due to privacy or ethical restrictions.
